# Spindle Cell Carcinoma of the Lung/Pleura: An Incidental Finding

**DOI:** 10.7759/cureus.2848

**Published:** 2018-06-20

**Authors:** Muhammad Sardar, Muhammad Azharuddin, Wahab J Khan, Mohammad A Noory, Nasreen Shaikh, Saad Ullah Malik, Doantrang Du

**Affiliations:** 1 Internal Medicine, Monmouth Medical Center, Long Branch, USA; 2 Pathology, Monmouth Medical Center, Long Branch, USA; 3 Hematology, University of Arizona, Tuscon, USA

**Keywords:** spindle cell carcinoma, sarcomatoid carcinoma

## Abstract

A 59-year-old male with a medical history of abdominal aortic dissection underwent a follow-up computed tomography (CT) scan abdomen, which showed an incidental pleural-based mass in the left lung base. The patient underwent an ultrasound (US)-guided biopsy and the histology was consistent with spindle cell carcinoma (SpCC). Staging workup was concerning for a metastatic lesion on the adrenal gland. The patient refused surgery and was subsequently started on chemotherapy. SpCC is a rare histological variant of sarcomatoid carcinoma. The prognosis is generally poor and treatment is the same as for other non-small cell lung cancers (NSCLC). The literature on disease progression and treatment is limited.

## Introduction

Sarcomatoid carcinoma (SC) of the lung are rare, comprising of a small subset of non–small cell lung cancer (NSCLC). According to the Surveillance, Epidemiology, and End Results (SEER) Database (1973-2013), SC was present in only 0.52% of all diagnosed cases of NSCLC [1]. SC is a heterogeneous group of tumors without any definite diagnostic criteria [2]. Based on histological features, it is classified into five major types by the World Health Organization (WHO), including pleomorphic carcinoma, spindle carcinoma, giant cell carcinoma, carcinosarcoma, and pulmonary blastoma [3]. Spindle cell carcinoma (SpCC) is defined as a rare histological type of SC, consisting of spindle-shaped tumor cells [4]. There is a paucity of data on clinical presentation, disease progression, and treatment of SpCC. We present a case of a 59-year-old male who was incidentally diagnosed with a lung mass (later proven to be SpCC), whose initial presentation was with symptoms unrelated to his final diagnosis. We believe this case will be a useful contribution to the literature as this type of cancer is rarely seen and even less frequently reported. 

## Case presentation

A 59-year-old African American male with a medical history of hypertension and abdominal aortic dissection presented to our medical clinic complaining of back pain for one month. He described it as dull, non-radiating, and with no associated motor or sensory weakness. The patient denied shortness of breath, chest pain, productive cough, weight loss, night sweats, or loss of appetite. Physical examination was unremarkable. The patient reported a 10-year pack smoking history. One year prior to the current presentation, he was admitted to our hospital with severe stabbing abdominal pain radiating to the back. Computed tomographic (CT) angiography was done which showed an abdominal aortic dissection extending from thoracic aorta to left common iliac artery that was managed conservatively with tight blood pressure control. The patient admitted to not having any follow-up imaging since discharge from the hospital.

**Figure 1:**
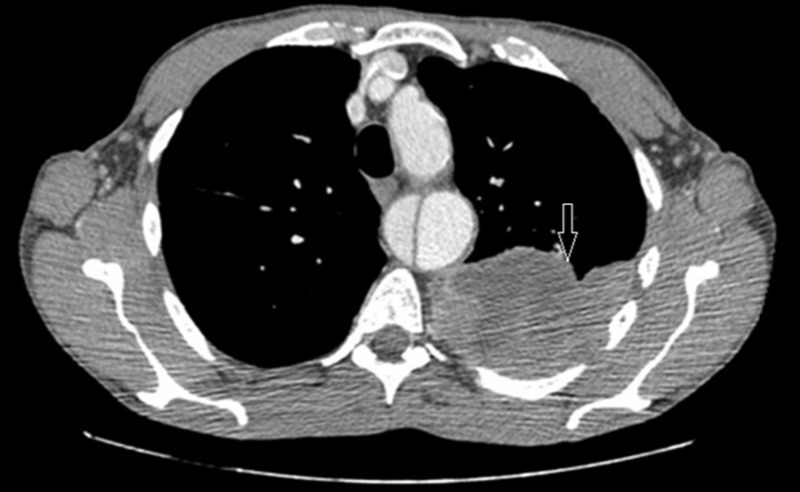
Computed tomography (CT) chest with contrast - axial section The arrow shows a 21.5 x 9 x 10.2 cm mass in the posterior mediastinum.

A CT scan of the abdomen and pelvis was ordered to evaluate the abdominal aortic dissection as the cause of his back pain. The results showed a stable long segment type B dissection of the descending thoracic aorta extending into the left common iliac artery. An incidental finding of a large lobulated pleural-based mass was also seen in the lower lobe of the left lung. A CT scan of the chest with contrast was ordered for better visualization of the mass, which again identified a large lobulated pleural-based mass in the posterior mediastinum measuring 21.5 x 9 x 10.2 cm (Figure 1).

**Figure 2:**
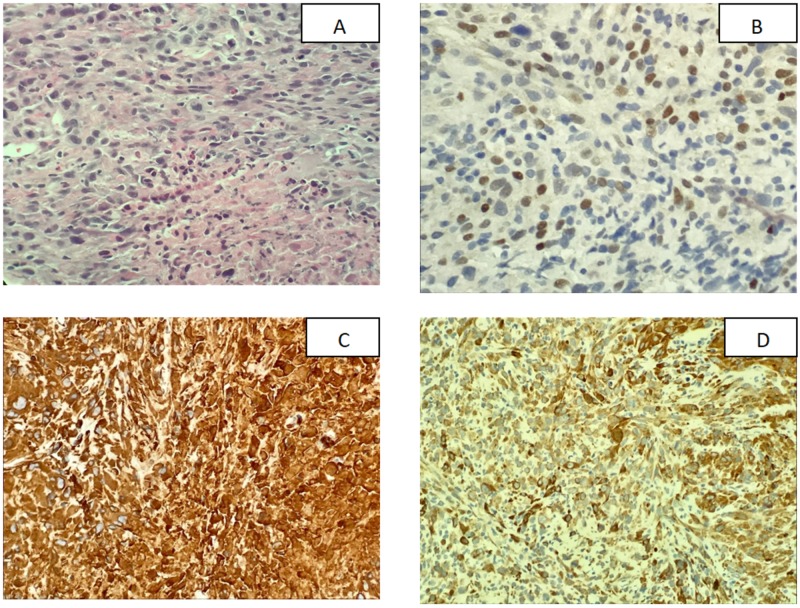
Histological analysis and immunohistochemical staining of the lung biopsy (A) Hemotoxylin and eosin staining showed that the tumor cells are predominantly composed of spindle cells with increased mitotic activity and areas of necrosis; (B) Tumor cells were weakly positive for P63 staining; (C) Vimentin showed strongly positive tumor cells; (D) Cytokeratin-Oscar showed tumor cells to be strongly positive

A US-guided biopsy of the lung mass was done which showed a poorly differentiated malignant neoplasm with a predominantly spindle cell pattern and epithelioid features, consistent with spindle cell carcinoma. Immunohistochemical (IHC) analysis was performed, which was positive for programmed death-ligand 1 (PD-L1) with a tumor proportion score (TPS) of 85 - 90% (Figure 2).

A bone scan and magnetic resonance imaging (MRI) of the abdomen and pelvis were done to determine the staging. A bone scan did not reveal any abnormal activity suggestive of osteoblastic metastatic disease. The MRI of the abdomen revealed a 2 cm x 2 cm left adrenal lesion suspicious for metastatic disease. The patient refused to undergo surgical evaluation for resection of the mass, so he was started on chemotherapy. So far, he has received one cycle of Carboplatin with a target area under the curve (AUC) of five, and Pemetrexed, 500 mg/m^2^. He was also started on Pembrolizumab, 200 mg intravenously (IV) to be given every third week, given the high-grade PD-L1 expression. The patient is scheduled for follow-up with a positron emission tomography (PET)-CT scan after completing his third cycle of chemotherapy.

## Discussion

Little is known about the clinical presentation of spindle cell carcinoma (SpCC). Our patient was incidentally diagnosed from a CT scan of the abdomen and did not have any symptoms pertinent to his diagnosis of SpCC. SpCC of the lung is more commonly seen as a peripheral lesion, which was consistent with the pleural-based lesion on the left lower lobe in our patient. Radiographically, SpCC lesions have been reported to have low-density regions in the center on CT scan, which was the case in our patient as well, having a low radiodensity of 5 Hounsfield units (HU) in the central region [5]. SpCC is diagnosed on biopsy by the appearance of heavily mitotic malignant spindle cells with hyperchromic nuclei and an absence of pleomorphic or giant cells. Immunohistochemistry (IHC) in SpCC has been described to show strong positive reactions for cytokeratin and vimentin and negative reactions for desmin, S100 protein, α-smooth muscle actin, and CD34 [5]. In addition, our patient was weakly positive for P63 as well, which has a fundamental role in the epithelial development and plays a role in a number of tumors of epithelial origin [6]. Seventy-five percent of the lung sarcomatoid cancers (pleomorphic, spindle cell, giant cell) are positive for PD-L1. PD-L1 expression confers a poor prognosis but, on the other hand, also makes the patient a candidate for targeted immunotherapy therapy [7]

Prognosis of SpCC is dismal. In the case series of 11 patients by Qi et al., only three patients out of 11 had a survival of more than two years [8]. For patients with sarcomatoid carcinoma (SC), the treatment approach in eligible patients with localized disease consists of surgical resection with neoadjuvant/adjuvant chemotherapy [9]. The choice of chemotherapy is based on the results of the Phase I/II KEYNOTE-021 trial in NSCLC patients, which consists of a combination of pemetrexed and carboplatin, in addition to pembrolizumab [10]. Our patient refused to undergo surgical evaluation and hence was started on chemotherapy.

## Conclusions

Spindle cell carcinoma (SpCC) is a very rare tumor with a poor prognosis. The literature on the treatment and disease progression is still very limited, and there is a need for publishing long-term follow-up of such patients.
